# Supplementary material for: Sulfane Sulfur Is an Intrinsic Signal for the Organic Peroxide Sensor OhrR of Pseudomonas aeruginosa
Source: Antioxidants (Basel). 2022 Aug 26;11(9):1667. doi: 10.3390/antiox11091667 (PMC9495516; doi:10.3390/antiox11091667)
Supplement: Supplementary file 1 [file antioxidants-11-01667-s001.zip › antioxidants-1852857-supplementary.pdf]

Supplemental Information

Sulfane sulfur is an intrinsic signal for the organic peroxide sensor OhrR of *Pseudomonas aeruginosa*

Huangwei Xu[1,†], Guanhua Xuan[1,†], Huaiwei Liu[1], Honglei Liu[1], Yongzhen Xia[1,*] and Luying Xun[1,2,*]

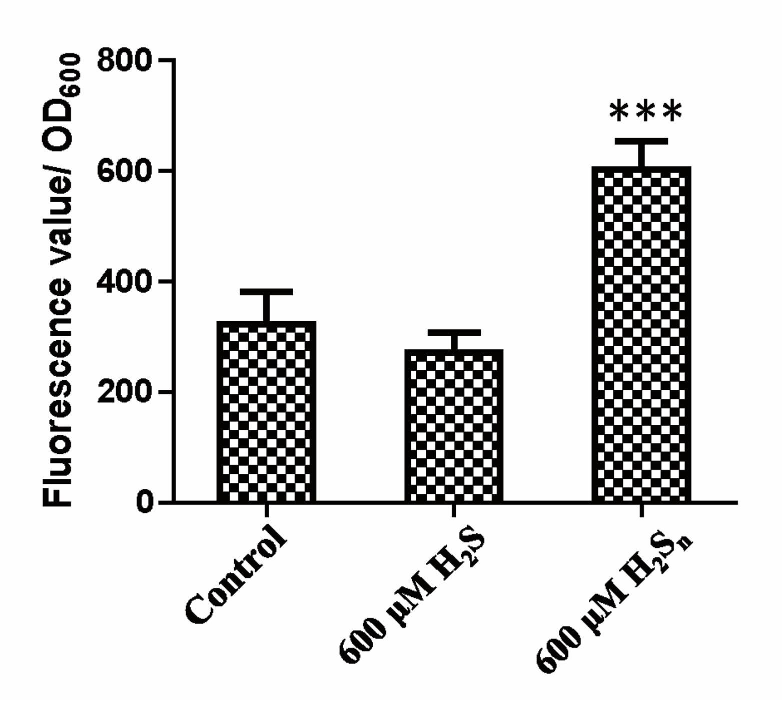

Figure S1. OhrR senses sulfane sulfur rather than H_2S. *E. coli* BL21 containing pBBR5-OhrR-P_{ohr}-mKate were induced by 600 μM H_2S or H_2S_n. The control was uninduced *E. coli*. Symbol *** indicate the sample is significantly different from the control ($p < 0.001$).

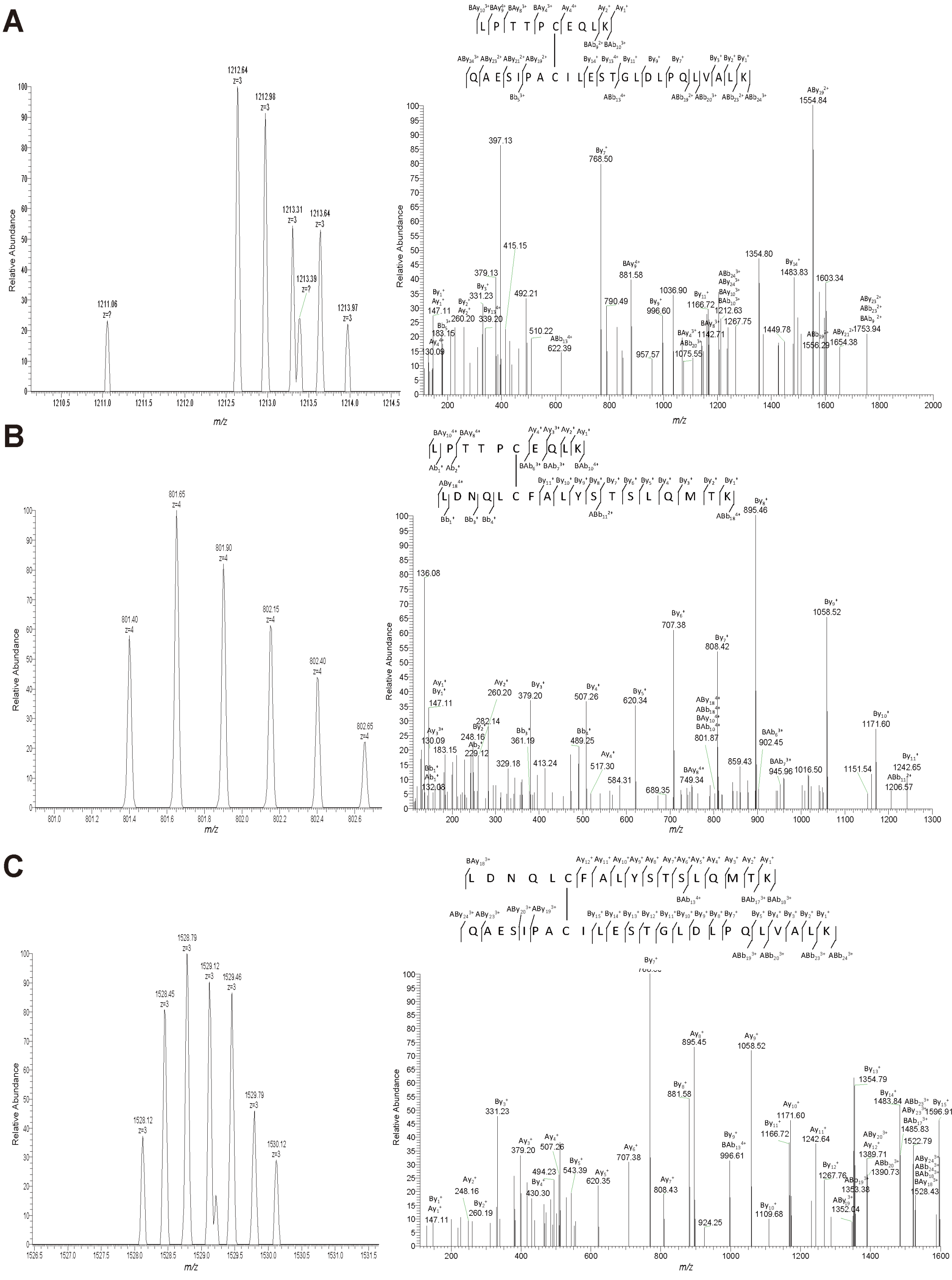

Figure S2. LC-MS/MS analysis of H_2S_n-treated OhrR. Mass spectrum of individual tryptic peptides of H_2S_n -treated OhrR. A) Left: The 3+ charged peak (m/z:1212.64) corresponding to the Cys^9-Cys^{121} disulfide-containing peptide of interest (theoretical molecular mass: 3635.93Da). Right: MS/MS fragmentation of the 3+ charged peptide (m/z: 1212.64). B) Left: The 4+ charged peak (m/z: 801.40)

corresponding to the Cys^{9}-Cys^{19} disulfide-containing peptide of interest (theoretical molecular mass: 3202.59 Da). Right: MS/MS fragmentation of the 4+ charged peptide (m/z: 801.40). C) Left: The 3+ charged peak (m/z: 1528.12) corresponding to the Cys^{19}-Cys^{121} disulfide-containing peptide of interest (theoretical molecular mass: 4582.35 Da). Right: MS/MS fragmentation of the 3+ charged peptide (m/z: 1528.12).

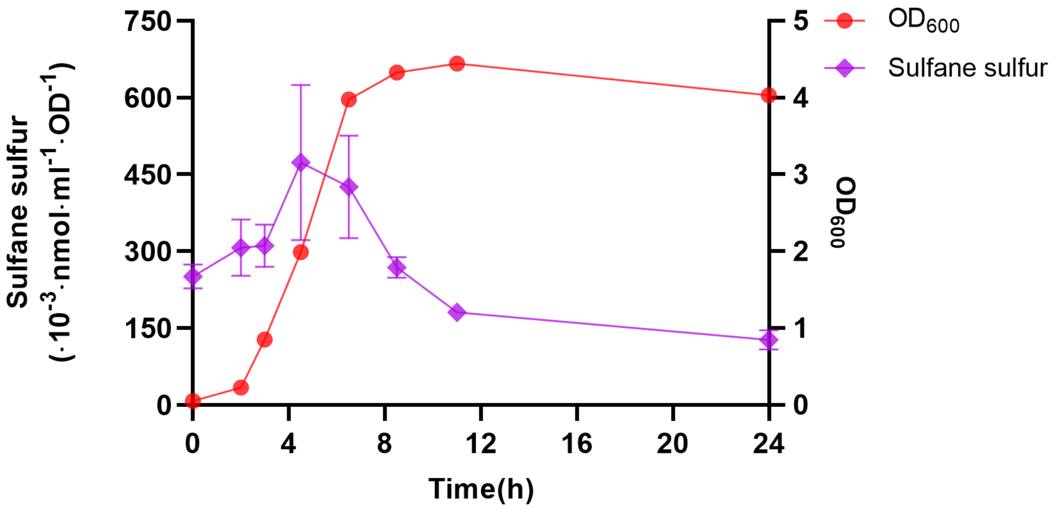

Figure S3. Intracellular sulfane sulfur of the reporter bacterium in LB medium changed with growth phases. Intracellular sulfane sulfur contents (■) and OD_{600nm} (●) were measured associated with growth phases. *E. coli* BL21 (pBBR5-OhrR-P*ohr*-mKate) was cultured in LB. The data are average of at least three samples with standard deviation (error bar).

Table S1. Strains and plasmids used in this study.

Strain/plasmid	Characteristic	Source
Escherichia coli BL21(DE3)	Cloning strain	Invitrogen
Pseudomonas aeruginosa P AO1	Wild type	ATCC 15692
Plasmids		
pBBR1mcs5	Gm, broad host range	Kovach [a]
pBBR5-OhrR –P_{ohr}-mKate	pBBR1mcs5 vector with *ohr* promotor, *ohrR,* and *mkate* genes	This study
pBBR5-OhrR –P_{ohr}-mKate- P_{trc}-pdo	pBBR1mcs5 vector with *ohr* promotor, *ohrR, mkate* and *pdo* genes	
pET28a	Kmr, expression vector	Invitrogen
pET28a-OhrR	pET28a containing OhrR with N terminal his-tag	This study
pET28a-OhrR/C19S	pET28-OhrR with Cys19Ser	This study
pET28a-OhrR/C121S	pET28-OhrR with Cys121Ser	This study
pET28a-OhrR/C19SC121S	pET28-OhrR with Cys19Ser, Cys121Ser	This study
pET28a-OhrR/C9S	pET28-OhrR with Cys9Ser, Cys19Ser	This study
pET28a-OhrR/C9SC19S	pET28-OhrR with Cys9Ser, Cys19Ser	This study
pET28a-OhrR/C9SC121S	pET28-OhrR with Cys9Ser, Cys121Ser	This study

Table S2. Primers used in this study.

Primers	Sequence (5'-3')	Usage
ohrR-1	AGCAAATGGGTCGCGGATCCATGTCCCGACTGCCCACCA	OhrR recombinant expression
ohrR-2	GGTGGTGGTGGTGCTCGAGTTCAATCCGGTGCTTGCAGGTTACC	
ohrR-C9S-1	CACCCCTTCCGAGCAGCTCAAGCTGGACAACCAG	OhrR/C9S recombinant expression
ohrR-C9S-2	GCTGCTCGGAAGGGGTGGTGGGCAGTCG	
ohrR-C19S-1	CCAGCTGTCCTTCGCCCTGTATTCCACCTCGCTG	OhrR/C19S recombinant expression
ohrR-C19S-2	GGGCGAAGGACAGCTGGTTGTCCAGCTTGAGCT	
ohrR-C121S-1	TCCCGCCTCCATCCTGGAGAGTACCGGCCTCG	OhrR/C121S recombinant expression
ohrR-C121S-2	CCAGGATGGAGGCGGGAATGCTCTCGGC	
ohrR+Pohr F	TCTAGAGAAAGAGGAGAAATACTAGATGTCCCGACTGCCCACCA	
ohrR+Pohr R	CTAGTATTTCTCCTCTTTCTCTAGAGAGAGTCACCTGTCTGATTTGTACGT	
mKate F	TCTAGAGAAAGAGGAGAAATACTAGATGTCAGAATTAATTAAAGAAAATATGCACATG	Primers for construction of pBBR5-OhrR-P_{ohr}-mKate
mKate R	CTTACAATTTCCATTCGCCATTTCAACGATGTCCTAATTTCGACG	
pBBR1MCS-5-F	CTAGTATTTCTCCTCTTTCTCTAGACAACATACGAGCCGGAAGCATAAAG	
pBBR1MCS-5-R	AATGGCGAATGGAAATTGTAAGCG	

[a] A ribosome binding site (sbs) sequence (gaggag) was inserted before *mkate*.

Table S3. Mass data of OhrR from LTQ-Orbitrap tandem mass spectrometry.

OhrR	Modification	Observed mass (MH^+)	Calculated mass (MH^+)
Peptide 1	R_9S-SR'$_{121}$	3635.9218	3635.9326
Peptide 2	R_9S-SR'$_{19}$	3202.6056	3202.5884
Peptide 3	R_{19}S-SR'$_{121}$	4582.3669	4582.3544

Peptide mass was calculated on the website:

http://db.systemsbiology.net:8080/proteomicsToolkit/FragIonServlet.html
